# Age estimation based on 3D post-mortem computed tomography images of mandible and femur using convolutional neural networks

**DOI:** 10.1371/journal.pone.0251388

**Published:** 2021-05-12

**Authors:** Cuong Van Pham, Su-Jin Lee, So-Yeon Kim, Sookyoung Lee, Soo-Hyung Kim, Hyung-Seok Kim

**Affiliations:** 1 Department of Electronics and Computer Engineering, Chonnam National University, Gwangju, South Korea; 2 Department of Forensic Medicine, Chonnam National University, Gwangju, South Korea; 3 Department of Forensic Medicine, National Forensic Service, Gangwondo, South Korea; 4 Department of Artificial Intelligence Convergence, Chonnam National University, Gwangju, South Korea; Vellore Institute of Technology: VIT University, INDIA

## Abstract

Age assessment has attracted increasing attention in the field of forensics. However, most existing works are laborious and requires domain-specific knowledge. Modern computing power makes it is possible to leverage massive amounts of data to produce more reliable results. Therefore, it is logical to use automated age estimation approaches to handle large datasets. In this study, a fully automated age prediction approach was proposed by assessing 3D mandible and femur scans using deep learning. A total of 814 post-mortem computed tomography scans from 619 men and 195 women, within the age range of 20–70, were collected from the National Forensic Service in South Korea. Multiple preprocessing steps were applied for each scan to normalize the image and perform intensity correction to create 3D voxels that represent these parts accurately. The accuracy of the proposed method was evaluated by 10-fold cross-validation. The initial cross-validation results illustrated the potential of the proposed method as it achieved a mean absolute error of 5.15 years with a concordance correlation coefficient of 0.80. The proposed approach is likely to be faster and potentially more reliable, which could be used for age assessment in the future.

## Introduction

Age assessment is one of the most active research fields in forensic and legal medicine. It can be used for some critical purposes, such as criminal identification [1], determining criminal responsibility through adult status prediction [2, 3], victim identification during natural disaster [4–6], or personal identification in the case of lacking legal documentation [7]. Despite some similarities among the employed methods, age estimation varies significantly for juveniles and adults regarding the specific techniques used as indicators. Juvenile age assessment is usually based on dental development characteristics and skeletal growth measurements [8]. By comparison, adult age prediction depends more on factors, such as the degeneration of particular bones, or even parts of the bones, namely ribs, cranial sutures, femur, or tibia. Most studies have argued that age assessment often performs more precisely for people who are still growing; in fact, various methods achieved promising results for juveniles. However, owing to the large age range corresponding to dental and skeletal maturity, proposing an appropriate method for adult age estimation is more challenging. To address this issue, it is believed that combining different features may lead to more precise and accurate results.

Many traditional child age estimations are based on the evaluation of dental development and skeletal growth. These methods depend on the eruption sequence of the teeth or developmental stage analysis using radiographs; among them, the most popular method is the Demirjian criteria [9, 10], which is based on the calcification of the permanent seven teeth on the left side of the mandible, and is considered to be the gold standard. The calcification of the tooth comprises eight stages, each of which is assigned with a particular score. Most studies based on this method used computed tomography (CT) scans of molars (particularly the first, second, and third) and premolars, owing to their more stable root morphology. Following the Demirijian’s criteria, Lee et al. [11] investigated the developmental stages of the second and third molars. Meanwhile, Aboshi et al. [12] used the mandibular molars and premolars to determine a ratio between pulp and tooth volume. In addition to the Demirijian’s criteria, another clavicle-based age assessment method has been presented. Yoon et al. [13] evaluated the age of adolescents and young adults using the ossification grade of medial clavicular epiphysis on chest radiographs; meanwhile, Rudolf et al. [14] studied the deformation of the anatomical shape of the sternum clavicle to obtain a more accurate age assessment. In addition to CT, magnetic resonance imaging (MRI) has been used frequently in many studies [15–17] owing to its lower radiation dose.

Adult age estimation could be more complicated owing to the lack of vital developmental markers, such as bone degeneration. Various studies have used dental radiographs [18–21] for age assessment based on the Kvaal method. However, the dental age alone may not be sufficient for accurate age predictions for adults. Age may also be estimated from the femur, which is less commonly used compared to the dental and skeletal structures. The study conducted by Rissech et al. [22] showed that the femur could play a key role in adult age prediction. Alunni et al. [23] also determined the sex and age by evaluating certain characteristics of the femur, particularly the femur head shape and bone densitometry. Moreover, several studies indicated that a combination of different features could lead to more precise and accurate results. Stern et al. [24] used both dental and skeletal MRI data in their studies. De Tobel et al. [25] combined multi-factorial agent indicators, including four third molars, the left wrist, and both clavicles. Kumagai et al. [26] used radiographic development, by combining permanent teeth with four skeletal predictors, to estimate the age of children. These studies achieved improved accuracy and promising results compared to approaches using single age indicators. Although they have also investigated the optimal combination manner of individual predictors for age assessment, it is still an open question owing to the lack of a corresponding standard.

However, the above-mentioned methods are usually based on regression, which commonly suffers from the drawback of being variable because of the time-consuming radiologic visual examination. An automatic end-to-end age estimation technique is necessary to overcome these drawbacks. The recent advances of deep learning (DL) have introduced significant benefits to medical image analysis in various areas, such as liver cancer segmentation, pneumothorax segmentation, skin lesion analysis toward melanoma detection, and autonomic cancer detection and classification [27]. In the age estimation field, DL-based methods have yielded some promising results. Guo et al. [28] utilized real-world low-quality X-ray images to successfully perform age estimation. Other studies, which were conducted by Stern et al. [24, 29–31], demonstrated that DL-based approaches could achieve an accuracy much higher than conventional methods, and even achieve expert-level performance for age estimation using teeth, clavicles, and hands. These results demonstrated the potential of DL, and encouraged further investigation of its usage in age estimation systems. To the best of our knowledge, there exists no DL-based method proposed for age prediction through the examination of both the mandible and femur.

In this study, a novel DL-based age estimation approach was presented via a combined analysis of both mandible and femur scans. Meanwhile, the corresponding contribution of different body parts on age has also been thoroughly investigated. The final obtained results confirmed the effectiveness and validity of the proposed approach for age estimation.

## Materials and methods

In this study, 814 whole-body (head to toes) post-mortem computed tomography (PMCT) scans were collected for the target experimental dataset. All scans were obtained from the National Forensic Service (NFS) in South Korea by NFS IRB approval(#906-190124-HR-005-02), and all data were fully anonymized before our access. The subjects consist of 619 males and 195 females, with an age range from 20 to 70 years old (mean ± std: 49.59 ± 11.80). As shown in Table 1, the majority of the test subjects belong to the age range between 40 and 60 years old, with the peak age range being 50–60.

**Table 1:** Gender and age distribution of the dataset.

Characteristics	Number of patients	Percentage (%)
Gender		
Male	619	76.04
Female	195	23.96
Age Group (years)		
20–30	68	8.35
31–40	113	13.88
41–50	207	25.43
51–60	269	33.05
61–70	157	19.29
Total	814	

Whole-body PMCT scans were collected for 814 people (619 males; 195 females), whose age ranged from 20 to 70 years old and were classified by age group.

We employed the exclusion criteria for evaluation presented by Lee et al. [32]. The subjects with mandible fractures, artifacts, head injuries, or jawbones damage were ignored. Each PMCT slice was encoded into digital imaging and communications in medicine (DICOM) format files, which are useful for integration among all modern imaging equipment and contain several unique features that are important for practicing radiologists.

### Proposed methods

#### 1. Processing steps

**Fig 1:**
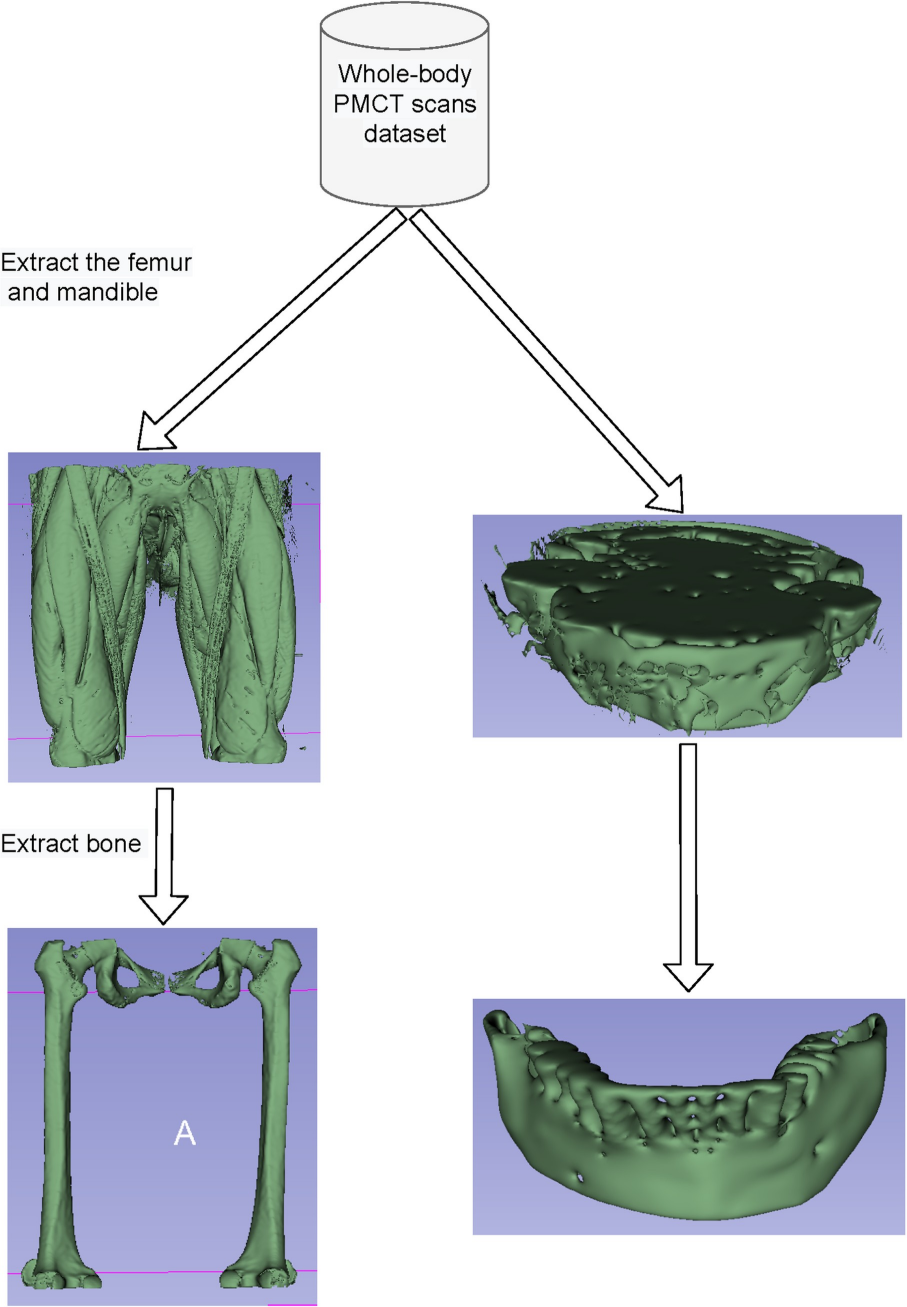
Preprocessing to extract the femur and mandible voxels. Initially, the femur and mandible parts were extracted from whole-body (head to toes) PMCT images, followed by segmentation of their corresponding bone parts.

Each folder in the dataset represents a scan (one patient), comprising multiple slices. From each scan, the mandibles and femurs were extracted as useful age indicators, which were subsequently preprocessed to create 3D voxels before being fed to the convolutional neural network (CNN) model, as shown in Fig 1.

We first loaded the DICOM files, and then, added any missing metadata; particularly, we added the slice thickness, that is, the pixel size in the Z direction, which was obtained from the DICOM file. The unit of measurement in PMCT scans is the Hounsfield Unit (HU), which is a measure of radiodensity. Thus, we converted HU to pixel values. Subsequently, we resampled to an isomorphic resolution to remove the scanner resolution. The slice thickness refers to the distance between consecutive slices (when viewing a 3D image as a collection of 2D slices), and varies between scans. For instance, a scan may have a pixel spacing of [1.2, 1.1, 1.1], which means that the distance between the slices is 1.2 mm. For a different scan, a pixel spacing may be [0.8, 1.12, 1.12]. This could introduce a challenge for the CNN. Therefore, in this study, each slice was resized to 1×1×1 *mm*^3^ voxel.

The final preprocessing step is bone segmentation and pixel normalization. Because the femur has an HU value between 400 and 1000, values beyond this range were considered as irrelevant. Therefore, the scans were clipped between 400 and 1000 HU to obtain the bone part. Subsequently, each scan was rescaled to a value between 0 and 1 (with 400 mappings to 0 and +1000 to 1), which was followed by the formation of femur voxels with a shape of 112×128×128.

**Fig 2:**
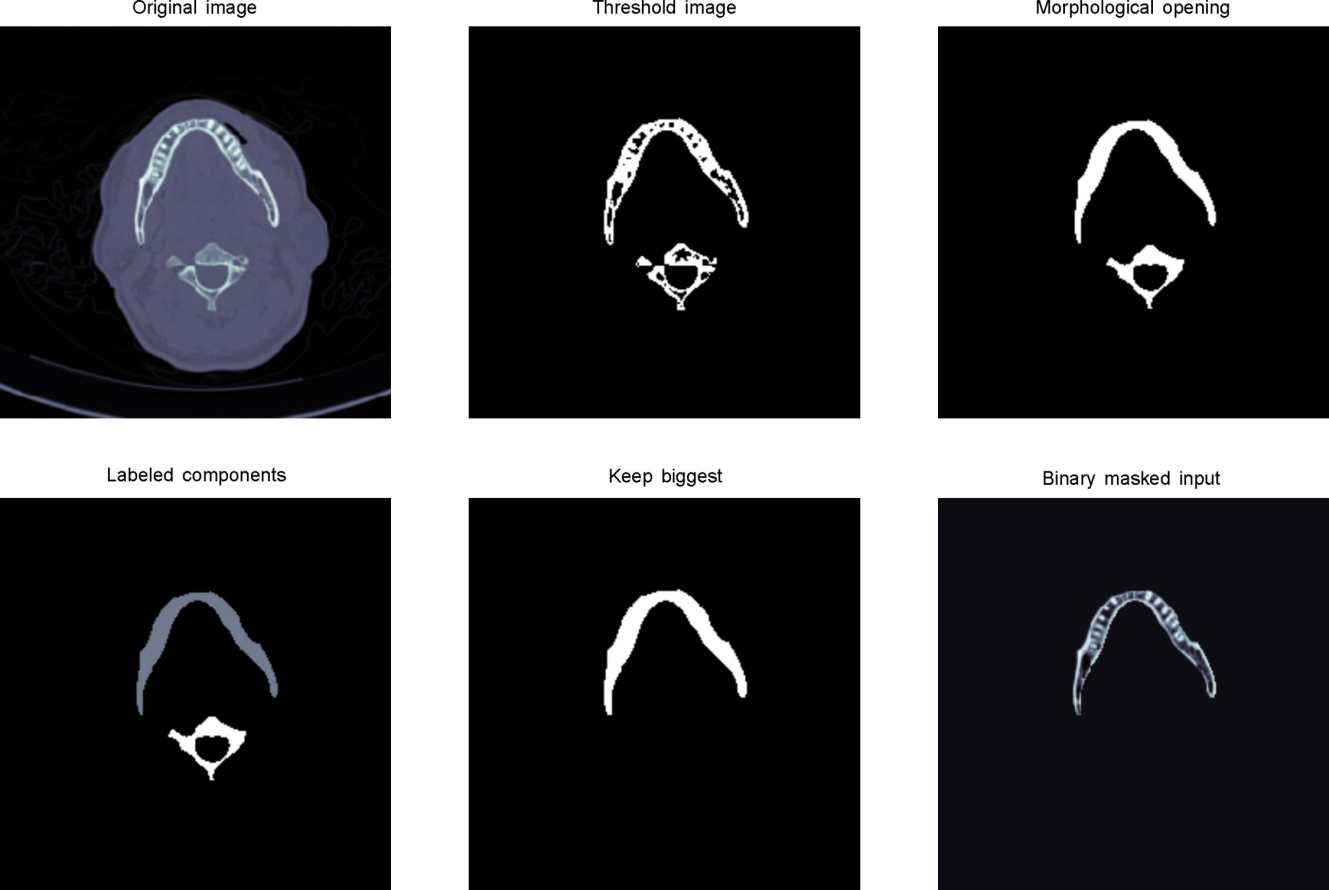
Mandible bone extraction from a raw slice image. (a) Load the original image and crop its center with an image size of 256×256. (b) Apply a threshold of 400 to remove tissue and unrelated parts. (c) Perform morphological opening operation, a technique to remove small objects, while maintaining the shape and property of large objects. (d) Label components to identify objects. (e) Retain the large objects in labeled components. (f) Binary masked input to obtain the bone part of mandible slice.

Mandible bone extraction is more complex because the neck bone has to be removed from the mandible. As shown in Fig 2, we applied image binary thresholding and morphological opening operation for each slice.

The morphological opening operation is an important technique in image processing, which is achieved by erosion, and then, the dilation of an image. This technique helps to remove small objects, while retaining larger parts, from an image [33]. To obtain the mandible bone part, we kept the largest areas after morphological opening. Finally, we stacked all the slices together to obtain the mandible voxels with the shape of 20×256×256.

#### 2. Overall framework

*2–1*. *Task description*. Age estimation can be defined as a regression problem. The input features for the framework can be considered as age indicators. In this study, the age indicators are the bone parts of the femur and mandible.

*2–2*. *CNN-based age estimation*. CNN model F will extract important features from input X (mandible or femur voxel).

$$feature_{X}=F_{\theta }\left(X\right)$$ (1)

where *θ* represents the parameters of the CNN models. Then, *feature*_*X*_ is fed into the fully connected (fc) layer to obtain the regression value.

$$\hat{y}=fc\left(feature_{X}\right)$$ (2)

We then acquire true age *y* = [*y*_1_,*y*_2_,*y*_3_,…,*y*_*n*_] and predicted age $\hat{y}=\left[{\hat{y}}_{1},{\hat{y}}_{2},{\hat{y}}_{3},\ldots ,{\hat{y}}_{n}\right]$. Our loss function is based on the mean squared error (MSE) between these ages, which can be expressed as follows:

$$MSE(y,\hat{y})=\frac{1}{n}\sum_{i=1}^{n}{\left(y_{i}-\hat{y_{i}}\right)}^{2}$$ (3)

**Fig 3:**
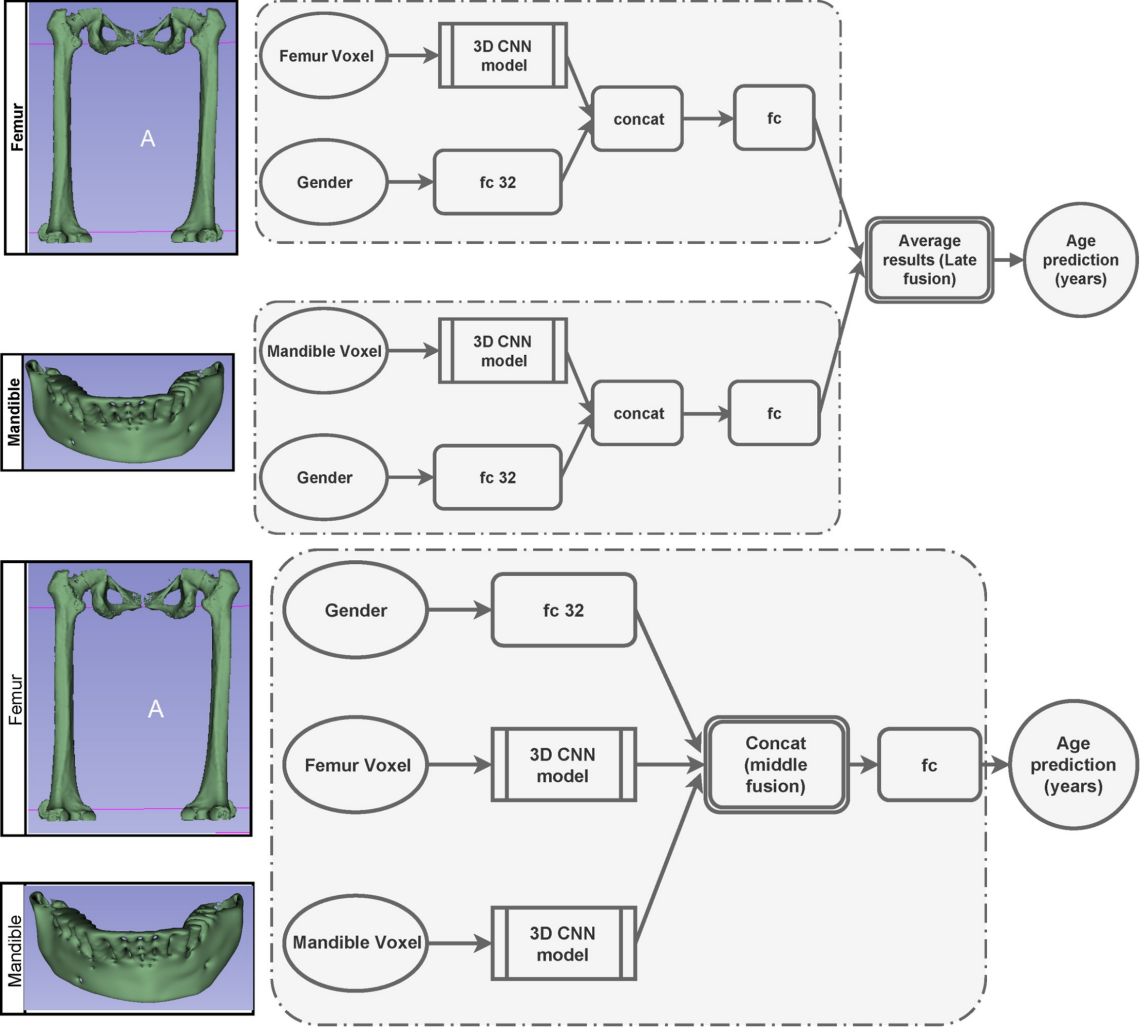
Overview of our age estimation architecture. 3D PMCT volumes of the femur and mandible are fed into the deep CNN, which then fuses their results to obtain the final precise prediction (years).

In terms of only femur or mandible as an input, we use gender features as our additional indicator. Therefore, we first extracted and combined the features with our gender network, which took binary gender information as input (0 for male and 1 for female), and then, fed it through a 32 fc network, as shown in Fig 3.

This concatenated layer is then fed through an fc layer. To achieve improvement by combing the mandible and femur, we applied the fusion technique. There exist three main fusion strategies. The first one is early modality fusion, in which different features are merged before being sent to the model. The second technique is middle fusion, where the features are projected by the models, and then, merged to obtain a combined feature. The third strategy is late fusion, in which independent models for each input are designed, and their predictions are combined via some form of model ensembling.

We decided to implement the late and middle fusion procedures, as shown in Fig 3. In case of late fusion, for each age prediction of mandible ${\hat{y}}_{\mathrm{mandible}}$ and femur ${\hat{y}}_{\mathrm{femur}}$, we averaged their results to produce final age estimation $\hat{y}$ (Fig 3A).

$$\hat{y}=\frac{1}{2}({\hat{y}}_{mandible}+{\hat{y}}_{femur})$$ (4)

In the case of middle fusion, we extracted the mandible and femur features after 3D CNN modeling, followed by their concatenation with gender features. These features were then fed into an fc layer to yield the final result, as shown in Fig 3B.

#### 3. Implementation details

**Fig 4:**
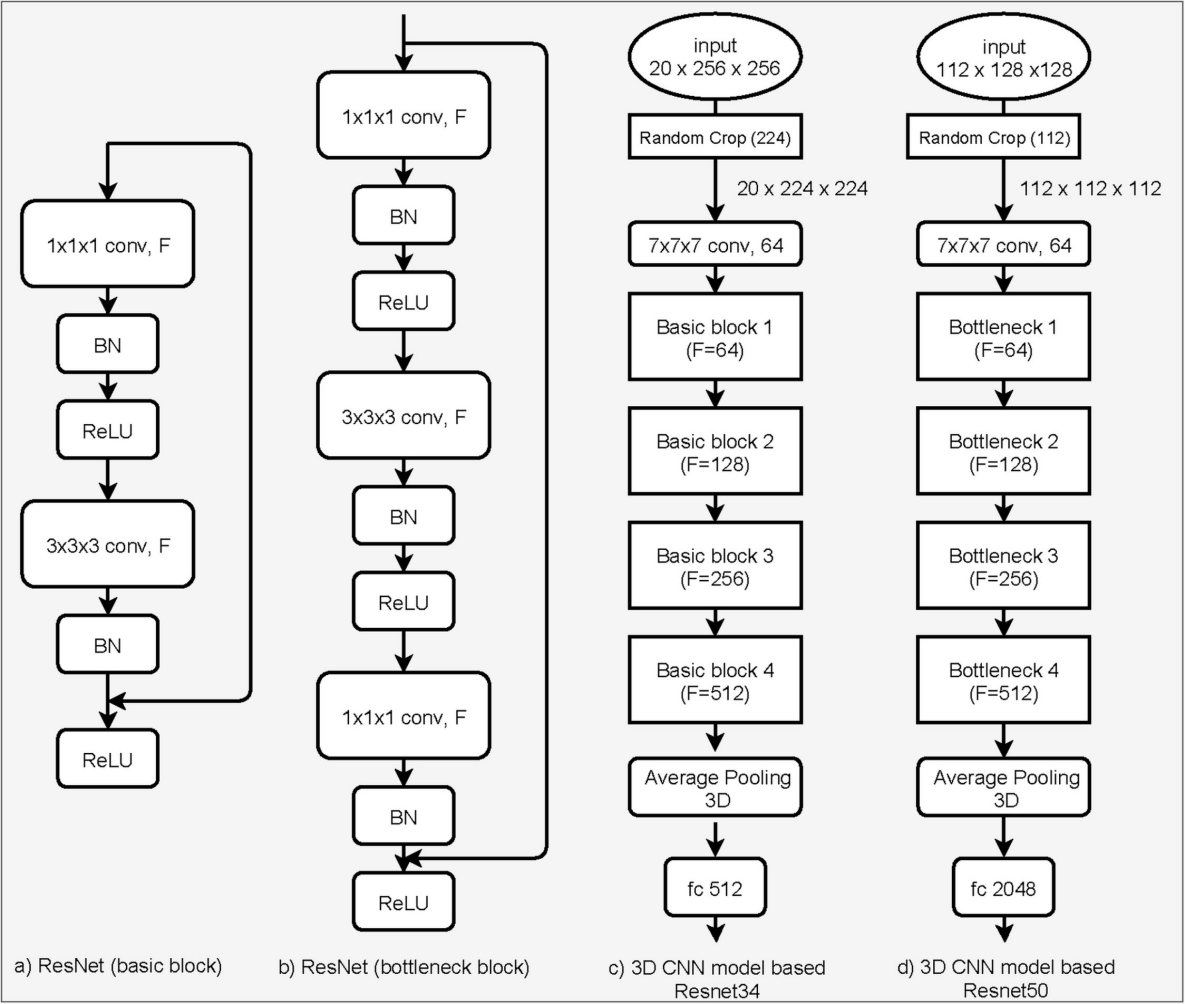
3D deep CNN architectures for age assessment. (a) Basic elements of the Resnet model, containing a convolutional layer with a number of filters (F), batch normalization (BN), and ReLU activation function; (b) bottleneck element of the Resnet model, which is an extension of the basic block by adding one more convolutional layer; (c) 3D CNN model based on Resnet 34, containing basic blocks with a different number of F; (d) 3D CNN model based on Resnet 50, including bottleneck blocks with a different number of F.

*3–1*. *Networks*. Recently, CNNs have been successfully applied in widespread medical image analysis and achieved significant benefits. We investigated the creation of a 3D CNN, with backbones based on Resnet [34], MobileNet [35], and SqueezeNet [36] models, which have proven to be the most efficient ones, and have been widely used in various applications. Our final architecture (see Fig 4) was based on 3D Resnet34 for the mandibles and 3D Resnet50 for the femurs.

The interesting part of Resnet is the "identity shortcut connection," which skips two CNN layers for the Resnet basic blocks and three CNN layers for the Resnet bottleneck blocks, as shown in Fig 4A and 4B, respectively. 3D Resnet34 comprises four Resnet basic blocks, while Resnet50 includes four Resnet bottleneck blocks, as shown in Fig 4C and 4D.

*3–2*. *Training*. For model training, we applied transfer learning and fine-tuning to all the models. To do this, all the models were first trained with smaller input sizes, and then, their weights were used as pre-trained models with larger input sizes. This significantly boosted the convergence time of models. We trained our model for up to 50 epochs. We used the Adam optimizer with a minibatch size of 16, weight decay of 1e-4, and momentum of 0.9 to decrease the mean absolute error (MAE) of the output. The learning rate started at 3e-4, with the minimum value being 3e-5. During the training procedure, the dynamic learning rate scheduling technique was used to reduce the learning rate by a factor of 0.8 after each 10 iterations, when the validation loss had plateaued. For data augmentation, we applied normalization, and randomly cropped the mandible and femur voxels to dimension scales of 20×224×224 and 112×112×112, respectively.

#### 4. Experimental setup

Regarding the validation method for age prediction in forensic anthropology, Andrea et al. [37] discussed common issues that were encountered when using incorrect procedures. Several reported studies did not validate over unseen data, which could lead to an overfitting problem. To solve such an issue, the k-fold cross-validation approach is an effective protocol for experimental validation with small samples. By using k-fold, we randomly split our dataset into *k* equal parts with the same distribution. We trained our model *k* times. For each model, we used a single partition from our *k* parts for testing and the rest for training. In our empirical experiments, we applied 10-fold cross-validation, which was considered the best choice in our case. The final accuracy was the average of all the obtained accuracies in each test set.

For the age estimation results, we computed the MAE, which is a common loss function used for regression models. It is the mean of the sum of absolute differences between our ground truth and predicted value, which is defined as follows (smaller is better).

$$MAE=\frac{1}{n}\sum_{i=1}^{n}|y_{i}-{\hat{y}}_{i}|=\frac{1}{n}\sum_{i=1}^{n}|y_{i}-f(x_{i})|$$ (5)

where *n* is the number of subjects, *y*_*i*_ is the ground truth of the ith sample, and ${\hat{y}}_{i}$ is the age predicted by model f, given the input feature *x*_*i*_.

Other popular measures for age estimation is the Pearson correlation coefficient (p) and concordance correlation coefficient (CCC) between the predicted ($\hat{y}$) and ground truth (*y*) ages, which are defined as follows.

$$\rho (y,\hat{y})=\frac{cov(y,\hat{y})}{\sigma_{y}*\sigma_{\hat{y}}}=\frac{E[\left(y-\mu_{y}\right)*\left(\hat{y}-\mu_{\hat{y}}\right)]}{\sigma_{y}*\sigma_{\hat{y}}}$$ (6)

$$CCC(y,\hat{y})=\frac{2\rho *\sigma_{y}*\sigma_{\hat{y}}}{\sigma_{y}^{2}+\sigma_{\hat{y}}^{2}+{\left(\mu_{y}-\mu_{\hat{y}}\right)}^{2}}$$ (7)

where *cov* is the covariance. *σ*_*y*_ and $\sigma_{\hat{y}}$ indicate the standard deviation of *y* and $\hat{y}$, respectively, while *μ*_*y*_ and $\mu_{\hat{y}}$ represent the mean of *y* and $\hat{y}$, respectively. E is the expectation.

## Results

By using our proposed approach, we investigated three different architectures with our dataset of 814 people in the 20–70 age group. As listed in Table 2, the first architecture used mandibles as an age indicator and produced a final MAE of 7.07 years; in comparison, using femurs produced a final MAE of 5.74 years.

**Table 2:** Age estimation results obtained based on the mandible and femur separately, and their corresponding fusion results using both male and female samples.

	Mandible		Femur		Late fusion		Early fusion		Middle fusion	
	MAE (year)	CCC	MAE (year)	CCC	MAE (year)	CCC	MAE (year)	CCC	MAE (year)	CCC
Fold 1	8.04	0.54	6.1	0.76	5.74	0.74	6.22	0.78	5.65	0.75
Fold 2	6.78	0.69	5.53	0.77	5.46	0.79	5.7	0.77	5.09	0.76
Fold 3	6.31	0.72	5.94	0.76	5.22	0.79	5.08	0.8	5.14	0.82
Fold 4	6.74	0.64	5.01	0.84	4.93	0.81	5.18	0.8	4.43	0.84
Fold 5	7.34	0.59	5.51	0.8	5.39	0.76	5.48	0.79	5.19	0.82
Fold 6	6.74	0.67	5.44	0.8	5.07	0.81	5.83	0.76	4.81	0.84
Fold 7	6.74	0.65	6.03	0.77	5.52	0.78	5.48	0.8	5.25	0.81
Fold 8	7.14	0.65	5.48	0.79	5.53	0.79	5.19	0.83	5.11	0.81
Fold 9	7.19	0.63	6.16	0.71	5.57	0.73	5.14	0.82	5.35	0.8
Fold 10	7.65	0.58	6.2	0.75	5.63	0.74	5.27	0.8	5.47	0.78
**Means ±std**	**7.07 ±0.51**	**0.64 ±0.05**	**5.74 ±0.04**	**0.78 ±0.04**	**5.41 ±0.26**	**0.78 ±0.03**	**5.46 ±0.37**	**0.8 ±0.02**	**5.15 ±0.34**	**0.8 ±0.03**

When estimated with the first architecture, both male and female samples produced a final MAE of 7.07 years; when using the jaw and femur as age indicators, a final MAE of 5.74 years was achieved.

Combining the femur and mandible using late fusion and early fusion resulted in an MAE of 5.41 and 5.46 years, respectively. Combining the femur and mandible to a single model using middle fusion resulted in an MAE of 5.15 years, which was a significant improvement. In terms of the CCC, we achieved a promising result of 0.8, 0.78, and 0.8 when using middle, late, and early fusion, respectively, whereas results for the mandible and femur alone were 0.64 and 0.78, respectively. These results verified the effectiveness of combining different age indicators to improve age estimation. When using the MAE metric, the standard deviation of 10 folds, when using the ensemble technique (late fusion and middle fusion), is far better than that obtained when using femur and mandible alone; the standard deviations were 0.26 and 0.34 for late and middle fusion, respectively. Meanwhile, the mandible yields the worst standard deviation, which was 0.51. In the case of the CCC metric, the mandible also obtained the highest standard deviation (0.05). By contrast, the standard deviation of all femur, late fusion, and middle fusion were 0.03. This proved the stability of using the ensemble technique.

We also evaluated the effectiveness of the gender features. In fact, owing to the small dataset size, we had to augment both the male and female samples. In our case, we have 685 male and 185 female samples. The female sample size is quite small for DL; therefore, we merely performed more experiments using the male samples with the same configuration. As shown in Table 3, when using middle fusion, we achieved the best accuracy in terms of MAE and CCC with corresponding means equal to 5.02 and 0.81, respectively.

**Table 3:** Age estimation results for mandible and femur alone, and their corresponding fusion results using only male samples.

	Mandible		Femur		Late fusion		Early fusion		Middle fusion	
	MAE (years)	CCC	MAE (years)	CCC	MAE (years)	CCC	MAE (years)	CCC	MAE (years)	CCC
Fold 1	7.0	0.61	4.0	0.87	5.5	0.74	4.41	0.84	3.89	0.87
Fold 2	6.37	0.64	5.7	0.75	6.04	0.7	5.1	0.8	4.83	0.82
Fold 3	7.36	0.53	5.43	0.8	6.39	0.67	4.53	0.84	4.91	0.81
Fold 4	6.82	0.62	6.41	0.67	6.62	0.65	6.15	0.69	5.25	0.78
Fold 5	7.03	0.55	5.97	0.72	6.5	0.64	5.36	0.78	5.84	0.75
Fold 6	7.07	0.6	4.61	0.84	5.84	0.72	4.69	0.84	4.58	0.81
Fold 7	6.83	0.68	4.94	0.84	5.88	0.76	5.03	0.84	5.06	0.81
Fold 8	7.1	0.62	5.6	0.79	6.35	0.71	5.16	0.8	4.9	0.84
Fold 9	6.85	0.66	6.54	0.65	6.7	0.66	5.66	0.77	5.81	0.76
Fold 10	7.27	0.57	4.92	0.8	6.09	0.69	5.55	0.74	5.1	0.81
**Means**	**6.97**	**0.61**	**5.41**	**0.77**	**6.19**	**0.69**	**5.164**	**0.79**	**5.02**	**0.81**

When only male samples were used, the means of MAE and CCC were 5.02 and 0.81, respectively, achieving the highest accuracy with middle fusion.

Further, the comparison of results from Resnet, MobileNet, and SqueezeNet for extracting femur features is presented in Table 4.

**Table 4:** Comparing age estimation results using SqueezeNet, MobileNet, and Resnet models for extracting femur features.

	3D Reset50		3D MobileNet		3D SqueezeNet		3D Reset50		3D MobileNet		3D SqueezeNet	
	MAE (years)	CCC	MAE (years)	CCC	MAE (years)	CCC	MAE (years)	CCC	MAE (years)	CCC	MAE (years)	CCC
Fold 1	6.1	0.76	6.19	0.74	9.07	0.29	0.87	4.0	6.1	0.65	8.33	0.31
Fold 2	5.53	0.77	6.93	0.65	8.19	0.5	0.75	5.7	6.21	0.69	7.52	0.46
Fold 3	5.94	0.76	5.99	0.69	8.43	0.34	0.8	5.43	5.29	0.78	8.87	0.28
Fold 4	5.01	0.84	6.33	0.71	8.42	0.38	0.67	6.41	6.81	0.64	8.3	0.34
Fold 5	5.51	0.8	6.98	0.68	7.66	0.52	0.72	5.97	6.75	0.64	7.81	0.36
Fold 6	5.44	0.8	5.89	0.73	7.11	0.56	0.84	4.61	5.97	0.74	7.94	0.37
Fold 7	6.03	0.77	5.77	0.76	7.96	0.41	0.84	4.94	6.14	0.74	7.26	0.5
Fold 8	5.48	0.79	6.9	0.65	7.36	0.58	0.79	5.6	6.71	0.74	7.83	0.48
Fold 9	6.16	0.71	5.92	0.78	7.88	0.43	0.65	6.54	7.08	0.63	8.47	0.46
Fold 10	6.2	0.75	8.63	0.22	7.2	0.65	0.8	4.92	6.77	0.6	6.5	0.58
Means	**5.74**	**0.78**	**6.55**	**0.66**	**7.93**	**0.47**	**0.77**	**5.41**	**6.38**	**0.69**	**7.88**	**0.41**

When using the entire dataset, 3D Resnet, MobileNet and SqueezeNet were used, with the 3D Resnet achieving the lowest MAE (5.74 years) and the lowest CCC (5.41), when using only male samples.

For our entire dataset, we achieved an MAE of 5.74, 6.55, and 7.93 years using 3D Resnet, MobileNet, and SqueezeNet, respectively, compared to the values of 5.41, 6.38, and 7.88 when using only male samples. From Tables 3 and 4, it can be observed that the results obtained using only male samples are often slightly higher than those obtained using both male and female samples. This indicates that a possible increase in our dataset size could contribute positively in helping our method achieve better results.

## Discussion

Inspired by the fact that there exists no standardized deep learning-based age assessment method, and with the aim of producing a faster, more precise, and more reliable age estimation, we proposed an end-to-end fully automated approach for the age assessment of adults from PMCT data. In this study, we investigated 814 adults aged between 20 and 70, which is a relatively large dataset with a wide age range. By using individual PMCT scans as input, our model can predict the age in only a few seconds. The proposed method achieved promising results, with an MAE of 5.15 years and a CCC of 0.80. The experimental results demonstrated that the MAE of the ensemble technique achieved better results than using only the mandible or femur for estimation, which supported the initial hypothesis of this study. We also found that the accuracy of using the femur alone was better than using the mandible alone in each fold, as well as in the overall results. This might be because the mandible has some associated bias effects during preprocessing. This also indicates that the femur could be a better age indicator for adults and should be further investigated.

There are no concrete recommendations that can be drawn on which method is better than the other. Bias can occur owing to several factors, such as data collection methods, different populations, the size of a dataset, the age range, and the method of analysis. Compared to other studies related to adult age estimation, our achieved accuracy was better than that reported in Zhang et al. [21] (5.15 years as against 8.81 years) in terms of MAE. Moreover, our best result (0.80) indicates higher accuracy with a similar age range to the research reported by Jagannathan et al. [38] (0.397), Aboshi et al. [12] (0.698), and Tardivo et al. [39] (0.47), in terms of the CCC. Their approaches reconstructed the dental structure, and then, calculated the pulp to enamel ratio to determine a regression equation for age assessment. These methods did not depend on a CNN and used a relatively small dataset. Our result (MAE of 5.15 years) was promising in forensic fields compared to that of Mathew et al. (MAE of 6.96 years) [40], and was within acceptable error limits for forensic age estimation (<±10 years) [41, 42]. Processing PMCT with our method could be practically beneficial, as it takes less than 1 min to estimate the age once the image is acquired. Moreover, because our approach is based on the DL technique, it can produce more sophisticated results as the training data size increases.

**Fig 5:**
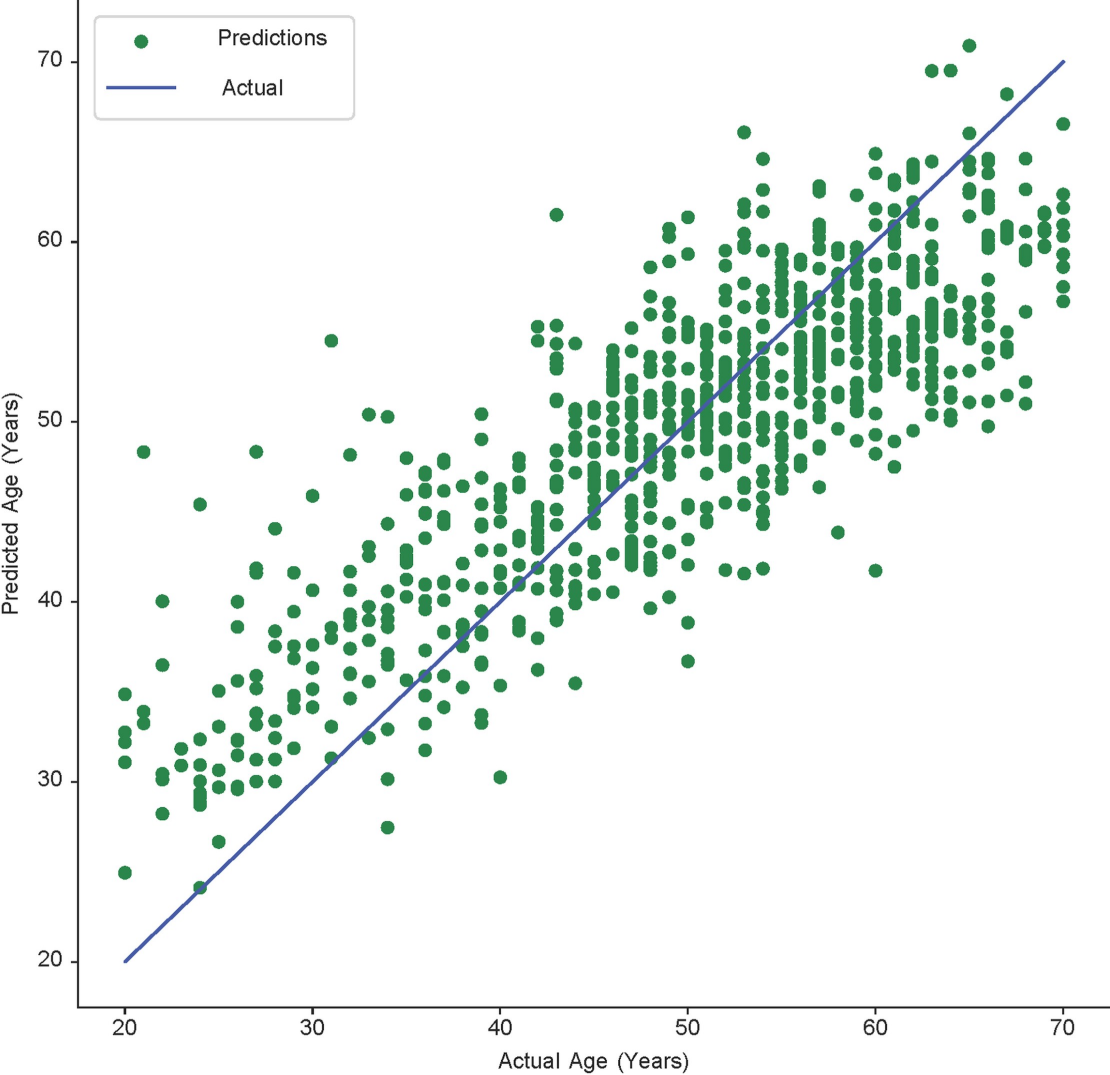
Scatter plot showing the final prediction results achieved by fusing the mandible and femur. This plot shows the correlation between the predicted and actual ages. The points follow a linear pattern, implying that there exists a high linear correlation. The increasing amount of green dots close to the linear line indicates the improving accuracy of the age estimation.

A significant limitation of our research is the data collection phase. The dataset used in this study was still insufficient and biased. The number of patients in the 40–60 age group was relatively large, while other groups were smaller, which could be responsible for obtaining better results for patients aged 40–60. As shown in Fig 5, several predicted ages are close to the actual age.

Additionally, owing to the limited sample size, we had to experiment with both males and females concurrently. Future studies should be conducted on each gender separately to gain deeper insights and to better evaluate the approach. Finally, those models were based on modern Korean people, and thus, should be validated over various populations in a larger sample. Finally, while traditional methods can be explained from statistical perspectives, interpreting a DL model is challenging; this could be regarded as a limitation of deep learning-based approaches, although they could lead to better results.

Another limitation of our work is the bone extraction accuracy from the femur and mandible. We applied a binary thresholding process to segment the mandible and femur regions from the entire scan, which consist of bone. In the case of femurs (as shown in Fig 1), we should remove the hipbone to decrease the bias. A similar issue happens during the mandible extraction; that is, a part of the neck may remain in the input after preprocessing. This problem could introduce a bias in the final result. Therefore, additional work on preprocessing the femur and mandible becomes necessary to overcome such bias. For that, we can employ a DL method to segment a mandible or femur from the PMCT scan, similar to the studies [43–46].

In conclusion, we have demonstrated that combining the PMCT scans of the femur and mandible as inputs to a 3D CNN could produce more accurate age estimation for an adult. We demonstrated the advantages of a deep learning-based method compared to traditional approaches, in terms of speed, accuracy, and reliability using a large dataset. An automated system is important for aiding the experts in determining the chronological age of a person based on their femur and mandible. We regard this work as the first step in producing a novel end-to-end automated method for age estimation using a 3D CNN model. Future work could cover topics such as the combination of more age indicators and their relevant segmentations before being employed for age estimation.
